# Loss of BRG1 induces CRC cell senescence by regulating p53/p21 pathway

**DOI:** 10.1038/cddis.2017.1

**Published:** 2017-02-09

**Authors:** Guihua Wang, Yinjia Fu, Fuqing Hu, Jinqing Lan, Feng Xu, Xi Yang, Xuelai Luo, Jing Wang, Junbo Hu

**Affiliations:** 1 Cancer Research Institute, Tongji Hospital, Huazhong University of Science and Technology, Wuhan, China; 2 Department of Immunology, Tongji Medical College, Huazhong University of Science and Technology, Wuhan, China

## Abstract

Brahma-related gene-1 (BRG1) is the specific ATPase of switch/sucrose nonfermentable chromatin-remodeling complex that is aberrantly expressed or mutated in various cancers. However, the exact role of BRG1 in oncogenesis remains unknown. In this study, we demonstrate that the knockdown (KD) of BRG1 promotes cellular senescence by influencing the SIRT1/p53/p21 signal axis in colorectal cancer (CRC). In particular, we reveal that the expression level of BRG1 is inversely correlated with p21, one of the classic senescence regulators, and is decreased in senescent CRC cells. KD of BRG1 promoting senescence is indicated by the increase of senescence-associated *β*-galactosidase (SA-*β*-gal) activity, inhibition of cell proliferation, induction of cell cycle arrest, and formation of senescence-associated heterochromatin foci. BRG1 binds to SIRT1 and interferes with SIRT1-mediated deacetylation of p53 at K382. Rescue experiments by co-silencing p53 or treatment with EX527, a SIRT1-specific inhibitor, abrogated the cellular senescence induced by KD of BRG1. BRG1 KD cells resulted in smaller tumor formation than that in control cells *in vivo*. Collectively, our study shows that BRG1 has an important role in cellular senescence and tumor growth. The BRG1/SIRT1/p53 signal axis is a novel mechanism of cell senescence in CRC and is a new potential target for cancer therapy.

Chromatin remodeling is one of the most important mechanisms regulating gene expression. The mammalian switch/sucrose nonfermentable (SWI/SNF) complex mediates the ATP-dependent chromatin remodeling and interacts with various transcriptional co-factors to manipulate gene expression.^1, 2^ Brahma-related gene-1 (BRG1) is a central subunit of the SWI/SNF complex that features ATPase activity involved in DNA repair, differentiation, and organ development.^3, 4^ BRG1 was reported as a tumor suppressor.^5^ BRG1 knockdown (KD) promoted tumor metastasis in colorectal cancer (CRC) via the miR-550/RNF43 pathway, indicating that BRG1 is a potential tumor suppressor.^6^ However, the role of BRG1 in cell senescence and proliferation remains controversial. BRG1 is upregulated and correlated with tumor proliferation in gastric cancer and contributes to acute myeloid leukemia maintenance.^7, 8^ Napolitano *et al.*^9^ and Kang *et al.*^10^ reported that Brg1 induces senescence and inhibits cell proliferation in rat mesenchymal stem cells and retinoblastoma, respectively. However, the role of BRG1 in colon cancer cell senescence and proliferation is not yet systematically explored.

Cancer as a disease is traditionally associated with immortal cells, and induction of cellular senescence leads to tumor regression.^11, 12, 13^ Impaired cellular senescence leads to enhanced tumor cell proliferation and promotes tumor progression.^14, 15^ Cell senescence is defined as a state of irreversible proliferative arrest that results in a senescent phenotype, which is characterized by upregulated senescence-associated *β*-galactosidase (SA-*β*-gal) activity, cell cycle arrest, formation of senescence-associated heterochromatin foci (SAHF), and inhibited cell proliferation.^13, 16^

P53 has an important role in cell senescence; acetylated p53 presents longer half-life and enhanced ability to activate p21 transcription, a master regulator of senescence.^17, 18, 19^ The P53/p21 pathway is a putative target for cancer therapy because of its significant effect on senescence in colon cancer, melanoma, glioma, and liver cancer.^20, 21, 22, 23^

In this study, we show that the expression level of BRG1 is inversely correlated with p21, and the KD of BRG1 promotes cellular senescence by increasing the SA-*β*-gal activity, inhibiting cell proliferation, inducing cell cycle arrest, and forming SAHF. We observed that BRG1 binds to SIRT1 and interferes with SIRT1-mediated deacetylation of p53 at K382. Rescue experiments by co-silencing p53 or treatment with EX527, a SIRT1-specific inhibitor, abrogated the cellular senescence induced by the KD of BRG1. BRG1 KD cells resulted in smaller tumor formation than that of control cells *in vivo*.

## Results

### Relationship between BRG1 and cellular senescence in CRC

**Figure 1:**
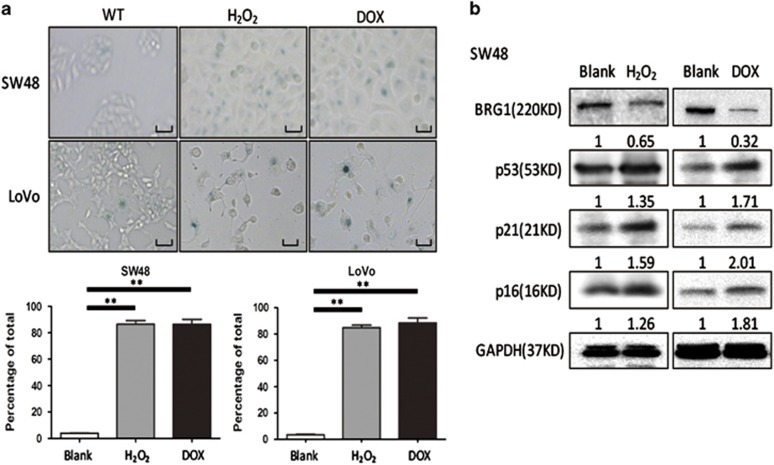
Relationship between BRG1 and cellular senescence in CRCs. (**a**) Cell senescence assay by SA-*β*-gal staining (up panel, representative images of SA-*β*-gal staining; scale bar: 50 *μ*m; down panel, percentage of SA-*β*-gal-positive cells from three random microscopic fields). (**b**) Western blot analysis for BRG1, p53, p21, and p16 expression levels in SW48 and LoVo cells treated with H_2_O_2_ (250 *μ*mol/l, 2 h) or DOX (1 *μ*mol/l, 48 h), **P<0.01

Senescence models induced by oxidative stress (H_2_O_2_) and DNA damage (doxorubicin, DOX) in colon cancer cell lines SW48 and LoVo were used to explore the role of BRG1 in cell senescence. SA-*β*-gal activity was examined to confirm the establishment of the senescence model induced by H_2_O_2_ (250 *μ*mol/l) and DOX (1 *μ*mol/l) (Figure 1a). We observed that the BRG1 expression significantly decreased in both senescent models (Figure 1b). These results revealed that low expression of BRG1 is related to the cellular senescence in CRC.

### Knockdown of BRG1 promotes cellular senescence in CRC cells

**Figure 2:**
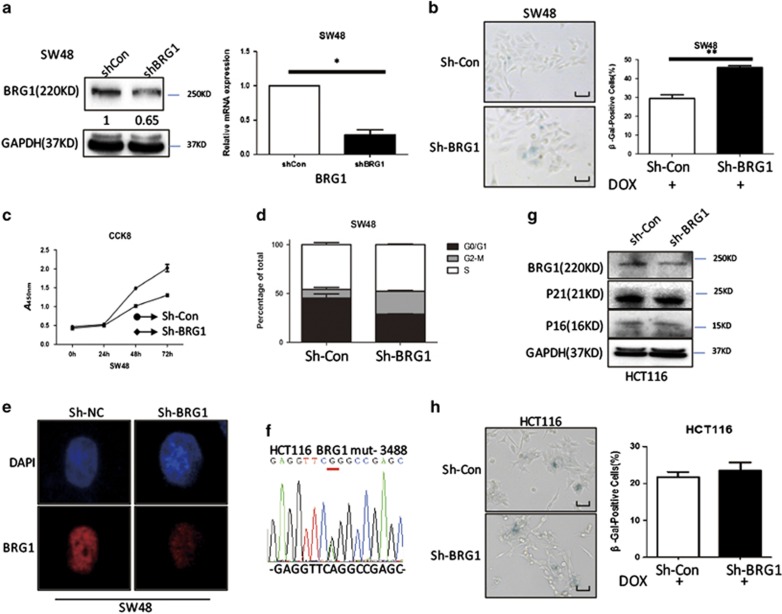
KD of BRG1 promotes cellular senescence in CRC cells. (**a**) Western blot and real-time-PCR analysis for BRG1 expression in SW48 Sh-Con and SW48 Sh-BRG1 cells. (**b**) Cell senescence assay by SA-*β*-gal staining (left panel, representative images of SA-*β*-gal staining; scale bar: 50 *μ*m; right panel, average counts of SA-*β*-gal-positive cells from three random microscopic fields) in SW48 Sh-Con and SW48 Sh-BRG1 cells. DOX (0.25 *μ*mol/l, 48 h). (**c**) Cell proliferation analysis determined by CCK8 assay (*n*=3, mean±S.D.) in SW48 Sh-Con and SW48 Sh-BRG1 cells (*n*=3, mean±S.D.; absorption at 450 nm (*A*450 nm) was, respectively, detected at 0, 24, 48, and 72 h after transfection). (**d**) Cell cycle distribution analysis measured by propodium iodide staining and flow cytometry in SW48 Sh-Con and SW48 Sh-BRG1 cells (*n*=3). (**e**) DAPI staining for SAHF formation in SW48 Sh-Con and SW48 Sh-BRG1 cells (red, BRG1; blue, DAPI. Magnification × 630). (**f**) DNA sequence analysis of HCT116 showing the mutation in BRG1 gene. (**g**) Western blot analysis for indicated protein expression levels in HCT116 Sh-Con and HCT116 Sh-BRG1 cells. (**h**) SA-*β*-gal staining analysis in HCT116 Sh-Con and HCT116 Sh-BRG1 cells. DOX (0.25 *μ*mol/l, 48 h); *P<0.05, **P<0.01, *t*-test

Cellular senescence is characterized by upregulated SA-*β*-gal activity, inhibited cell proliferation, cell cycle arrest, and formation of SAHF.^13^ A BRG1 KD cell model was produced by transfecting CRC cells with BRG1 shRNA (sh-BRG1) to further investigate the function and mechanism of BRG1 in senescence. Validation of sh-BRG1 led to a significant BRG1 KD in both protein and mRNA levels compared with control cells (sh-Con) (Figure 2a). In this study, we performed SA-*β*-gal assays with or without DOX treatment (0.25 *μ*mol/l). Data showed that the percentages of SA-*β*-gal-positive cells in SW48 sh-BRG1 cells significantly increased compared with sh-Con cells in both DOX treatment and non-treatment conditions (Figure 2b and Supplementary Figure 1). CCK8 assay was performed to evaluate the effect of BRG1 on cell proliferation. The results showed that the growth rates significantly decreased in BRG1 KD groups, suggesting that BRG1 KD inhibited cell proliferation (Figure 2c). Cell cycle analysis by flow cytometry detection was performed, and the results revealed that BRG1 KD led to cell cycle arrest at G2-M phase (Figure 2d). Furthermore, we examined SAHF by staining 4,6-diamidino-2-phenylindole (DAPI), which showed distinct SAHF formation in BRG1 KD cells as observed by laser confocal fluorescence microscopy scanning (Figure 2e). Annexin V assay revealed that BRG1 exerts no significant effect on apoptosis in CRC (Supplementary Figure 2). Previous experiments were also performed in HCT116 containing a BRG1 missense mutation to further verify the role of BRG1 in cell proliferation and senescence (Figure 2f). BRG1 reduction in HCT116 cells produced no significant alteration in SA-*β*-gal activity and expression of senescence protein markers compared with control cells (Figures 2g and h). A wild-type BRG1 was re-expressed in HCT116 cells, senescence-related protein expression was analyzed, and SA-*β*-gal activity assay was performed to further confirm the role of BRG1 in senescence. Data revealed that HCT116 cells undergo DOX-induced senescence when a wild-type BRG1 is re-expressed (Supplementary Figures 3a and 3b). Data also showed that another pathway mediates senescence in HCT116 cells without the BRG1 pathway. All these data revealed that BRG1 KD inhibits CRC cell proliferation by activating cellular senescence.

### Knockdown of BRG1 increases the expression level of p21

**Figure 3:**
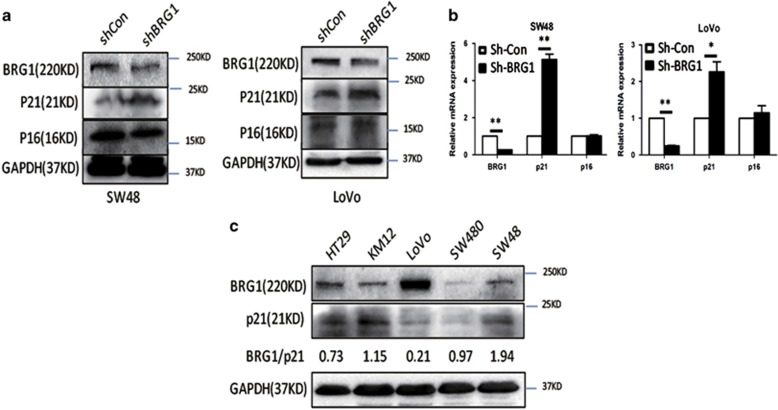
KD of BRG1 increases the expression level of p53 and p21. (**a** and **b**) Western blot analysis for expression levels of BRG1, p21, and p16, and quantitative expression analyses of BRG1, p21, and p16 mRNA by quantitative real-time PCR (*n*=3, mean±S.D.) in SW48 and LoVo Sh-Con and HCT116 Sh-BRG1 cells. (**c**) Western blot detected the expression levels of BRG1 and p21 in multiple colon cancer cell lines. BRG/p21: the relative expression ratio between BRG1 and p21. *P<0.05; **P<0.01

p21 and p16 are the most important signal factors that regulate senescence.^24^ Given that p21 and p16 are identified as the most important signal factors mastering in senescence, the effect of BRG1 on these pathways was determined to explore the senescence regulation of BRG1. BRG1 KD induced the upregulation of p21 compared with control cells but did not significantly affect the expression of p16 (Figure 3a). Real-time PCR assay results also showed upregulation of p21 mRNA expression in BRG1 KD cells but no change in p16 (Figure 3b). The expression levels of BRG1 and p21 were evaluated by western blot in five common CRC cell lines (HT29, KM12, LoVo, SW480, and SW48) to further confirm the correlation between BRG1 and the expression of p21. Data showed that p53 is wild-type in SW48 and LoVo, whereas BRG1 presents no significant correlation with p21 in the rest of the cell lines where p53 is mutated (Figure 3c and Supplementary Table 1). The expression levels of BRG1 and p21 were further detected and normalized by IHC staining in the primary tumor of the clinical specimens (*n*=36). The results revealed that BRG1 expression was inversely related with p21 expression (Table 1).

### BRG1 regulates the expression of p21 and cellular senescence dependent on regulation of p53 protein stability

**Figure 4:**
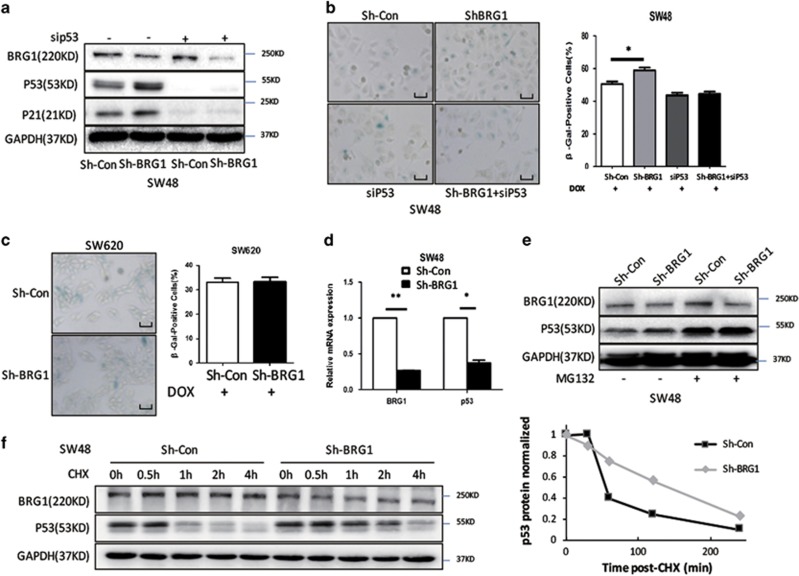
BRG1 regulates the expression of p21 and cellular senescence dependent on regulating p53 protein stability. (**a**) Western blot detected the expression levels of BRG1, p53, and p21 in SW48 Sh-Con and SW48 Sh-BRG1 cells. (**b**) SA-*β*-gal staining (left panel, representative images of SA-*β*-gal staining; scale bar: 50 *μ*m; right panel, percentage of SA-*β*-gal-positive cells from three random microscopic fields) in SW48 Sh-Con and SW48 Sh-BRG1 cells transfected with or without siP53. DOX (0.25 *μ*mol/l, 48 h). (**c**) SA-*β*-gal staining in SW620 transfected Sh-Con and Sh-BRG1 cells DOX (0.25 *μ*mol/l, 48 h). (**d**) Quantitative expression analysis of p53 mRNA by quantitative real-time PCR. (**e**) Western blot assays for BRG1 and p53 expression in SW48 Sh-Con and Sh-BRG1 cells treated with MG132(1 mmol/l). (**f**) Western blot assays for BRG1 and p53 expression in SW48 Sh-Con and Sh-BRG1 cells treated with CHX (1 *μ*g/ml) (up panel: western blot bands; down panel: plot of the p53 expression levels following CHX treatment in sh-Con cells or sh-BRG1 cells, normalized for the levels of GAPDH). *P<0.05; **P<0.01

p53 is the main transcription factor that regulates p21 expression.^20^ A group of ‘rescue' experiments were used by transfecting p53 siRNA in SW48 Sh-Con and Sh-BRG1 cells to confirm the dependence of BRG1 p21 regulation on p53. Our data showed that the increase of p21 induced by BRG1 KD was abrogated by the treatment of p53 siRNA (Figure 4a). Moreover, SA-*β*-gal-positive cells stopped increasing after the KD of BRG1 with p53 siRNA in both DOX treatment and non-treatment conditions (Figure 4b and Supplementary Figure 4). In SW620 and HT29, *β*-gal assays were repeated in two cell lines with p53 loss-of-function mutation. As expected, no significant changes were observed in SW620 and HT29 cells with BRG1 KD compared with the corresponding control cells (Figure 4c and Supplementary Figures 5a and 5b). The regulation of BRG1 in the p53/p21 pathway was further confirmed by using another shRNA against BRG1 (Supplementary Figure 6). These results suggested that KD of BRG1 promotes senescence by activating the p53 pathway in CRC cells.

The effect of BRG1 on p53 mRNA expression was investigated to identify the mechanism of BRG1 regulation in the p53 pathway and to further rule out the possibility that BRG1 transcriptionally regulates p53. Interestingly, we found that BRG1 KD did not increase; on the contrary, p53 mRNA expression decreased (Figure 4d). We further checked the post-transcriptional regulation of p53 in BRG1 KD condition because p53 protein function is also regulated at post-transcriptional level. We knocked down BRG1 with shRNA and treated the cells with proteasome inhibitor MG132 or cycloheximide (CHX), an inhibitor of protein biosynthesis. KD of BRG1 did not increase the p53 protein expression in cells treated with MG132 (Figure 4e). Conversely, upregulated p53 stability was observed in BRG1 KD cells with CHX treatment (Figure 4f). These results confirmed that BRG1 regulates p53 at a post-transcriptional level.

### BRG1 binding to SIRT1 and regulation of p53 acetylation

**Figure 5:**
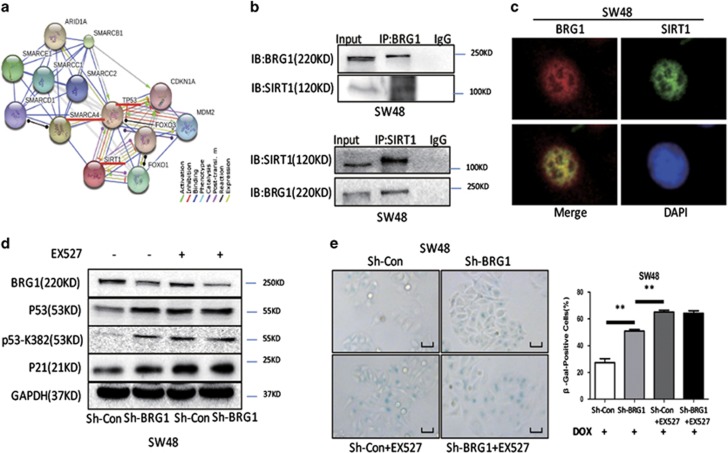
BRG1 binds to SIRT1 and regulates p53 acetylation. (**a**) The protein–protein networks view from STRING database showing the networks of BRG1 (SMARCA4, the gene name coding BRG1), p53, and SIRT1. (**b**) Co-immunoprecipitation analysis in SW48 cells using anti-SIRT1 antibodies and anti-mouse-IgG antibodies or anti-BRG1 antibodies and anti-mouse-IgG antibodies, respectively. (**c**) Immunofluorescence images scanned by confocal laser microscope detecting the sub-cellular location of BRG1 and SIRT1 expression levels in SW48 cells. (**d**) Western blot detected the expression levels of BRG1, p53, p53-K382, and p21 in SW48 Sh-Con and Sh-BRG1 cells treated with or without EX527 (1 *μ*mol/l, 48 h). (**e**) SA-*β*-gal staining (left panel, representative images of SA-*β*-gal staining; scale bar: 50 *μ*m; right panel, percentage of SA-*β*-gal-positive cells from three random microscopic fields) in SW48 Sh-Con and Sh-BRG1 cells treated with or without EX527 (1 *μ*mol/l, 48 h) DOX (0.25 *μ*mol/l, 48 h). **P<0.01

p53 acetylation is an important post-transcriptional modification that regulates p53 protein stability.^17, 25^ In addition, SIRT1 regulates p53 by deacetylating p53 at K382.^26, 27^ Deacetylated p53 shows decreased protein stability and transcriptional activity on p21 and is vulnerable to degradation.^18^ As indicated in the STRING Database (string-db.org) analysis, BRG1 (SMARCA4, the gene name of BRG1) directly interacts with SIRT1 (Figure 5a). Co-immunoprecipitation analysis showed that BRG1 binds to SIRT1 (Figure 5b). Protein colocalization was detected by laser confocal fluorescence microscopy scanning, and the results confirmed the interaction of BRG1 and SIRT1 (Figure 5c). SIRT1 inhibits p53 expression by deacetylating p53 at K382. Thus, we proposed a hypothesis indicating that BRG1 enhances SIRT1-mediated deacetylation of p53 at K382 via binding interaction. To confirm this hypothesis, we first examined the expression of SIRT1 in BRG1 KD cells and found that BRG1 does not affect SIRT1 expression (Supplementary Figure 7). We then analyzed the expression of K382-acetylated p53. BRG1 KD significantly increased the K382-acetylated p53 expression compared with the total p53 expression (data not shown). Subsequently, we performed a group of ‘rescue' experiments by treating cells with EX527, a specific function inhibitor of SIRT1 on p53-K382 deacetylation. The rescue experiments were performed to confirm that BRG1 regulates the p53/p21 pathway by manipulating the deacetylation effect of SIRT1. First, we confirmed the specificity of EX527 in SIRT1 by using SIRT1 shRNA (Supplementary Figure 7). The data of the following ‘rescue' assays showed that the increases of K382-acetylated p53 and total p53 induced by BRG1 KD were abrogated in the presence of EX527 (Figure 5d). The increase of SA-*β*-gal-positive cells caused by the KD of BRG1 was also abrogated in cells treated with EX527 (Figure 5e and Supplementary Figure 8). These results revealed that BRG1 synergizes the SIRT1 effect on p53 deacetylation at K382.

### Knockdown of BRG1 promotes senescence and inhibits cell proliferation *in vivo*

**Figure 6:**
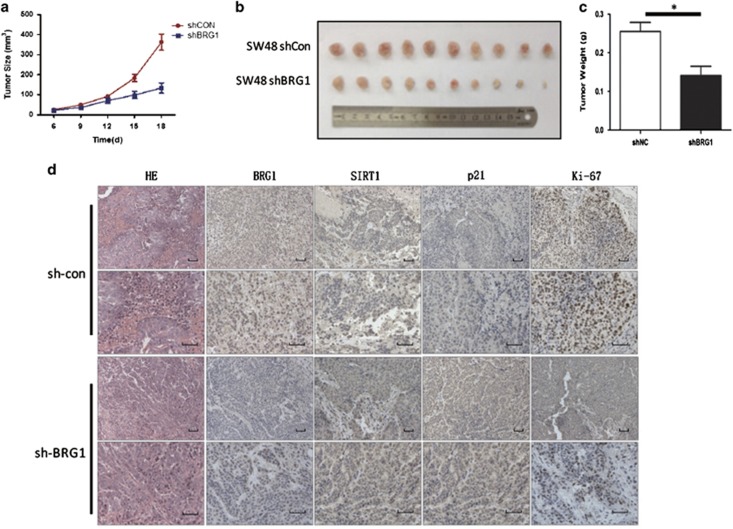
BRG1 KD promotes senescence and inhibits cell proliferation *in vivo*. (**a**) Tumor size measured as methods and material, and the tumor growth curve is shown. (**b**) All mice were killed 2 weeks after injection and the tumors were collected and shown. Box plot shows the distribution of tumor weight from sh-BRG1 *versus* sh-Con group (*n*=10, mean±S.D.). (**c**) Immunohistochemical analysis of BRG1, SIRT1, p21, and Ki-67 in tumor sections (scale bar: 50 *μ*m). (**d**) HE staining and IHC staining of indicated protein in xenograft. *P<0.05

SW48 sh-BRG1 cells and the corresponding negative control cells (SW48 sh-Con) were subcutaneously injected into nude mice (~1 × 10^6^ cells per mouse) to further evaluate the function of BRG1 in cell senescence and proliferation *in vivo*. We calculated the tumor volume every 2 days as the tumors became visible to naked eyes, and the volumes were plotted into a tumor growth curve as shown in Figure 5a. When all the mice were killed 2 weeks after injection, a significant difference of tumor volume between the sh-BRG1 and control groups was observed (Figure 6a). When all the mice were killed 2 weeks after cell injection, the weight of tumor formed by sh-BRG1 cells was 0.141±0.0763 g, whereas the weight in the control group was 0.255±0.0738 g (Figures 6b and c). Immunohistochemical analysis of the formed tumors showed decreased expression of BRG1 in the sh-BRG1 group compared with the control group, indicating that BRG1 KD is maintained *in vivo*. Moreover, an increased expression of p21 and reduced number of Ki-67-positive cells were observed in tumors formed by sh-BRG1 cells, and this finding suggests activated senescence and reduced proliferation. No difference on SIRT1 expression was found between the sh-BRG1 and control groups (Figure 6d). These results revealed that BRG1 KD induces cell senescence and inhibits CRC proliferation *in vivo*.

## Discussion

BRG1 is the specific ATPase of the SWI/SNF chromatin-remodeling complex that is correlated with oncogenesis of several kinds of cancers.^28^ However, the exact function and mechanism of BRG1 in tumorigenesis remain unclear, including its effect on cell senescence. BRG1 is mutated or aberrantly expressed in some kinds of tumors.^28^ In our previous study, we observed that BRG1 expression was reduced in metastatic tissues of CRC, indicating that BRG1 expression dynamically fluctuates by tumor progression.^6^ In this study, we found that BRG1 was inversely correlated with senescence in CRC cell lines in classic senescent cell models. Our data showed that the KD of BRG1 promoted colon cancer cell senescence, as indicated by the upregulation of SA-*β*-Gal activity, which led to cell cycle arrest, SANF formation, and inhibition of cell proliferation. This result conforms to the earlier reports of Hsu *et al.*^29^ and Naidu *et al.*^30^ but is opposite with the results in rat mesenchymal stem cell and retinoblastoma.^4^ A universal conclusion regarding the effect of BRG1 on cell senescence is difficult to achieve because cell models in these previous studies ranged from fibroblasts to stem cells. Given the diverse etiologic background of different tumors, BRG1 may have distinct roles in different cancer types. Although the correlation between BRG1 and senescence was previously reported, our study is the first to systematically explore the effect of BRG1 on senescence in CRC.

p21 and p16 are the most important signal factors that regulate senescence.^24^ In our study, BRG1 activated the p21 but exerted no effect on the p16 pathway in CRC cell lines conducted in clinical specimens. BRG1 expression was inversely correlated with p21, a master regulator of cell senescence, suggesting that p21 is a potential target of BRG1-regulating senescence. Our rescue experiments with siP53 in BRG1 KD SW48 cells or performed in SW620 and HT29 cells where p53 is mutated further confirmed that the KD of BRG1 promotes colon cancer cell senescence depending on the p53/p21 pathway. The upregulation of the p53 protein level by the KD of BRG1 suggested that BRG1 regulates p53 in a post-transcriptional manner. Interestingly, we also observed that BRG1 KD reduced p53 mRNA. In our experiments with MG132 or CHX, we confirmed that BRG1 regulates p53 by influencing its protein degradation. In particular, the KD of BRG1 enhanced p53 protein stability and produced longer half-life time. On the basis of recent theories, this BRG1-regulating mechanism for p53 is consistent with the classic p53 regulation.

SIRT1 is a mammalian NAD^+^-dependent deacetylase that is also referred to as class III histone deacetylases; this deacetylase is involved in several tumor progressions, such as melanoma, breast cancer, and CRC, via inhibition of cell proliferation.^31, 32^ SIRT1 exerts multiple effects on cellular metabolism, DNA repair, and senescence by deacetylating a variety of un-histone proteins, including p53. SIRT1 downregulates p53 expression at a post-transcriptional level by deacetylating p53 at K382.^32, 33^ Thus, SIRT1 executes its senescence regulation by inhibiting the p53/p21 pathway and subsequently promoting tumorgenesis.^34^ Cheng *et al.*^35^ reported that SIRT1 is highly expressed in CRC and inversely correlated with CRC prognosis, indicating that SIRT1 is a novel oncogene that induces CRC tumorigenesis. As expected, BRG1 KD upregulated the expression of K382-acetylated p53. In the rescue experiments with EX527, a specific inhibitor of SIRT1's function to deacetylate p53 at K382, we confirmed that BRG1 regulates CRC cell senescence mediated by SIRT1. In summary, we reported that the reduced BRG1 expression promotes senescence by interfering the SIRT1/p53/p21 signal axis to inhibit tumor proliferation in CRC.

Although BRG1, a central subunit of the SWI/SNF complex featuring ATPase activity, is involved in DNA repair, differentiation, and organ development, the effect of BRG1 on tumorigenesis and cancer cell senescence remains controversial. In this study, we showed that the BRG1/SIRT1/p53 signal axis is a novel mechanism that regulates CRC cell senescence and proliferation. Our animal assay showed that BRG1 KD inhibited the CRC proliferation by promoting senescence *in vivo*. The results revealed the importance of the BRG1/SIRT1/p53 signal axis in CRC tumorigenesis, which is also a potential target for a new strategy against cancer.

## Materials and methods

### Ethics statement

All studies involving human subjects were approved by the Huazhong University of Science and Technology (HUST) Ethics Committee, and formal consent was obtained from each subject. CRC specimens were derived from Tongji Hospital, HUST, Wuhan, China. All animal experiments were performed in accordance with the animal study guidelines of HUST.

### Cell lines and cell culture

All CRC cell lines (SW48, LoVo, SW480, HCT116, KM12, and HT29) were obtained from the American Type Culture Collection (ATCC, Manassas, VA, USA). The p53 status of all these cell lines was analyzed by COSMIC database (http://cancer.sanger.ac.uk/cell_lines) (Supplementary Table 1). All cells were cultured at 5% CO_2_ and 37 °C in Dulbecco's modified Eagle's medium that is supplemented with 10% fetal bovine serum (Thermo Fisher Scientific, Shanghai, China). Cells were treated with DOX (1 *μ*mol/l) or H_2_O_2_ (1 *μ*mol/l) following the recently published instruction to establish the cellular senescent model.^36^

### Antibodies and chemical materials

The antibodies to BRG1, p53, p21, p16, Rb, p-Rb, MDM2, and GAPDH were purchased from Santa Cruz Company (Santa Cruz, CA, USA), and the antibody to acetylated p53 at K382 was obtained from Abcam Company (Abcam, MA, USA). ADM, EX527, and MG132 were purchased from Selleck Company (Selleck Chemicals, Shanghai, China).

### IHC analysis

IHC analysis was simultaneously conducted by two pathologists using a multiple viewing microscope to evaluate the staining of BRG1 and p21. The quantification of IHC staining was evaluated by an IRS system. BRG1 and p21 staining patterns were determined as low (IRS: 0–4) and high (IRS: 6–12).

### RNA interference and lentiviral system

RNA interference targeting BRG1 and p53, as well as nonsense control siRNA sequence were synthesized and purified by RiboBio (Ribobio Co., Ltd, Guangzhou, China). siRNAs were transfected with Lipofectamine 2000 (Invitrogen, Carlsbad, CA, USA) following the manufacturer's guidelines. Lentivirus-expressing BRG1-targeted short hairpin RNA (sh-BRG1) and control lentivirus (sh-Con) was previously constructed in our laboratory.

### Senescence assays

SA-*β*-gal activity was measured following the manufacturer's instructions (Beyotime Biotechnology Ltd, Shanghai, China). SA-*β*-gal activity was examined by X-gal (5-bromo-4-chloro-3-3indolyl *β*-d-galactoside) staining at pH 6.0. Randomly selected fields (*n*=3) were analyzed by light microscope to quantify the percentage of SA-*β*-gal-positive cells. Cells were treated with DOX (0.25 *μ*mol/l) through SA-*β*-gal assays as previously described to produce DNA damage.

### Cell proliferation and cell cycle analysis

Cell proliferation was determined by CCK8 assays. Approximately 5000 cells were plated into each well of a 96-well plate and treated with corresponding processes. CCK8 solution (5 mg/ml) (Promoter Ltd, Wuhan, China) was added into each well for 3 h. The absorption of each well was then detected at 450 nm.

For cell cycle analysis, cells were fixed in 80% ethanol overnight at −20 °C, washed with phosphate-buffered saline, and then stained with propidium iodide and 100 *μ*g/ml RNaseA. DNA content was measured by sorting the fluorescence-activated cells on a Becton-Dickinson FACScan System (Franklin Lakes, NJ, USA).

### Immunofluorescence analysis

Cells were fixed and treated with 0.25% Triton X-100 for 10 min and incubated with anti-BRG1 and anti-SIRT1 antibodies at 4 °C overnight. Cells were washed with Tris-buffered saline-Tween (TBS-T), incubated with Dyelight 488 and Dyelight 549 for 30 min, and stained with DAPI. Fluorescent images were acquired through a confocal laser scanning system LSM510META (Carl Zeiss AG, Jena, Germany).

### Western blot analysis and real-time PCR assay

Western blot analysis was conducted as previously described.^37^ For co-immunoprecipitation experiments, Protein A/G PLUS-Agarose from Santa Cruz Company was used following the manufacturer's instructions. For real-time PCR, total mRNA was extracted using RNAiso plus (Takara Biotechnology, Dalian, China), and reverse transcription was performed with PrimeScript RT Master Mix (Takara Biotechnology). Real-time PCR was performed with Applied Biosystem 7300 (Thermo Fisher Scientific) by using SYBR Green Mix (Thermo Fisher Scientific).

The following primers were used: BRG1, 5′-CAGATCCGTCACAGGCAAAAT-3′ (forward) and 5′-TCTCGATCCGCTCGTTCTCTT-3′ (reverse); p21, 5′-TGTCCGTCAGAACCCATGC-3′ (forward) and 5′-AAAGTCGAAGTTCCATCGCTC-3′ (reverse); p16, 5′-GATCCAGGTGGGTAGAAGGTC-3′ (forward) and 5′-CCCCTGCAAACTTCGTCCT-3′ (reverse); p53, 5′-CAGCACATGACGGAGGTTGT-3′ (forward) and 5′-TCATCCAAATACTCCACACGC-3′ (reverse); SIRT1, 5′-TAGCCTTGTCAGATAAGGAAGGA-3′ (forward) and 5′-ACAGCTTCACAGTCAACTTTGT-3′ (reverse); and MDM2, 5′-GAATCATCGGACTCAGGTACATC-3′ (forward) and 5′-TCTGTCTCACTAATTGCTCTCCT-3′ (reverse).

### Animal study

Four-week-old female nude mice (Huafukang Biotechnology Ltd, Beijing, China) were housed in sterile filter-capped cages. Approximately 1 × 10^6^ cells suspended in culture medium were subcutaneously injected into the mice. The weight of tumors was measured and analyzed by paired *t*-test.

### Statistical analysis

All statistical analyses were conducted using SPSS13.0 (SPSS Inc, Chicago, IL, USA) statistical software package. Data were presented as mean±S.D., and two-tailed Student's *t*-test was performed using the data tools provided by the software. Statistical significance was considered at *P*<0.05.

## Figures and Tables

**Table 1:** The relationship of expression of BRG1 and p21 (Fisher's exact test)

	P21			
	H	L	Total	*P-*value
*BRG1*				
H	5	15	20	0.041
	13.9%	41.7%	55.6%	
L	10	6	16	
	27.8%	16.7%	44.4%	
Total	15	21	36	
	41.7%	58.3%	100%	
