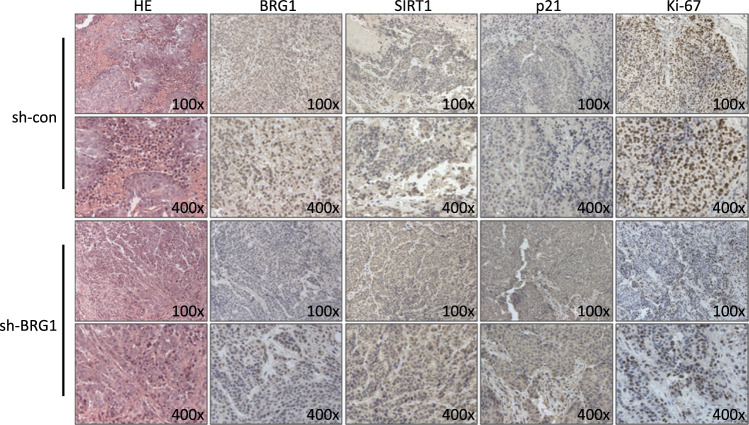# Correction: Loss of BRG1 induces CRC cell senescence by regulating p53/p21 pathway

**DOI:** 10.1038/s41419-021-04195-5

**Published:** 2021-10-01

**Authors:** Guihua Wang, Yinjia Fu, Fuqing Hu, Jinqing Lan, Feng Xu, Xi Yang, Xuelai Luo, Jing Wang, Junbo Hu

**Affiliations:** 1 Cancer Research Institute, Tongji Hospital, Huazhong University of Science and Technology, Wuhan, China; 2 Department of Immunology, Tongji Medical College, Huazhong University of Science and Technology, Wuhan, China

Correction to: *Cell Death and Disease* (2017) **8**: e2607; 10.1038/cddis.2017.1; published online 09 February 2017

Since online publication of this article the authors noticed there was an error in Fig. 6d. Incorrect images were used for the p21 and Ki-67 HE staining in the sh-BRG1 group. The figure has been corrected in the online html and PDF. The authors apologize for the error and confirm that these errors do not affect the results or conclusions of the paper.