# Supplementary material for: Loss of BRG1 induces CRC cell senescence by regulating p53/p21 pathway
Source: Cell Death Dis. 2017 Feb 9;8(2):e2607–. doi: 10.1038/cddis.2017.1 (PMC5386468; doi:10.1038/cddis.2017.1)
Supplement: Supplementary Figures [file cddis20171x1.ppt]

## Slide 1
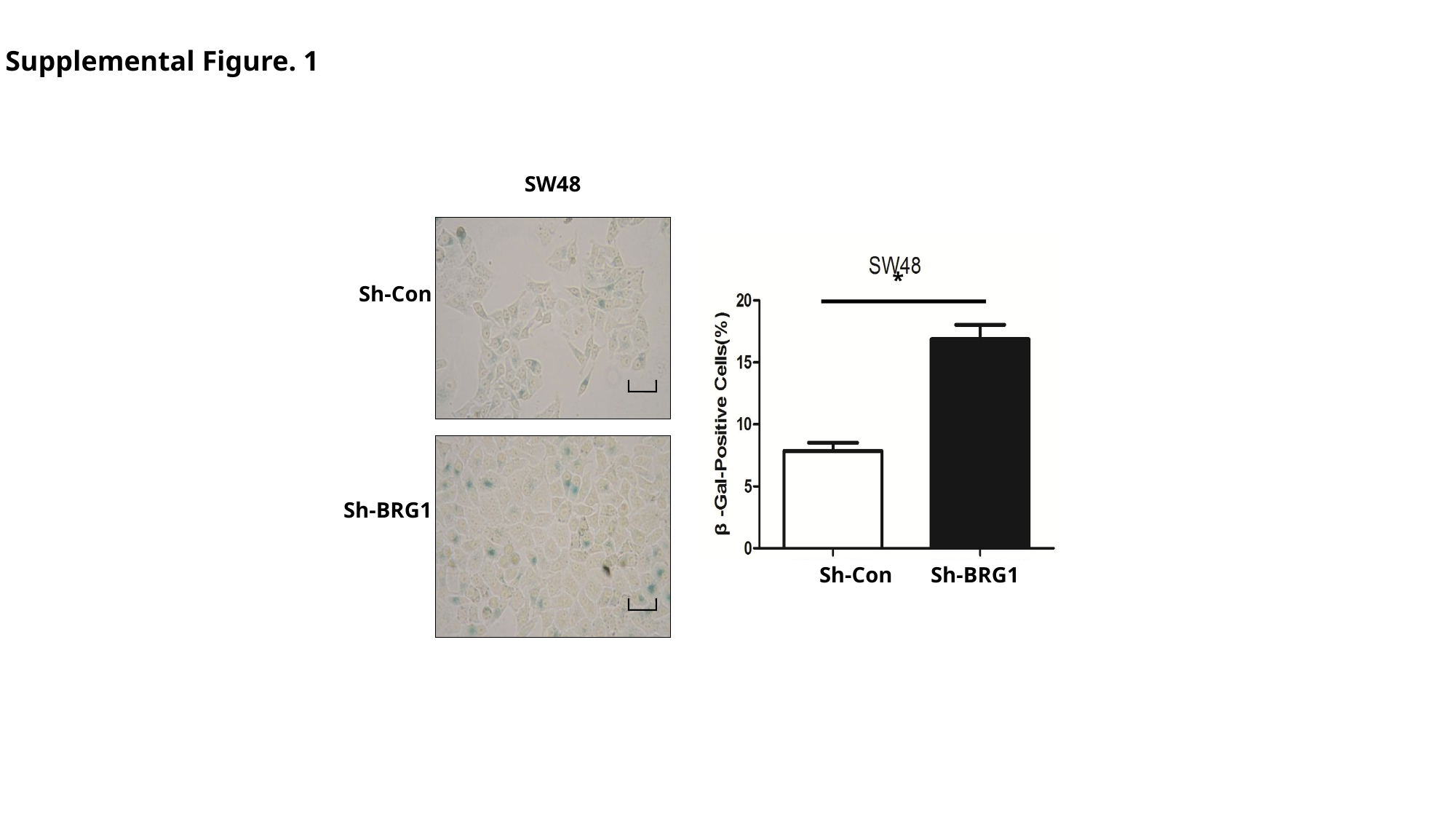

Supplemental Figure. 1
SW48
*
Sh-Con Sh-BRG1
Sh-Con
Sh-BRG1

## Slide 2
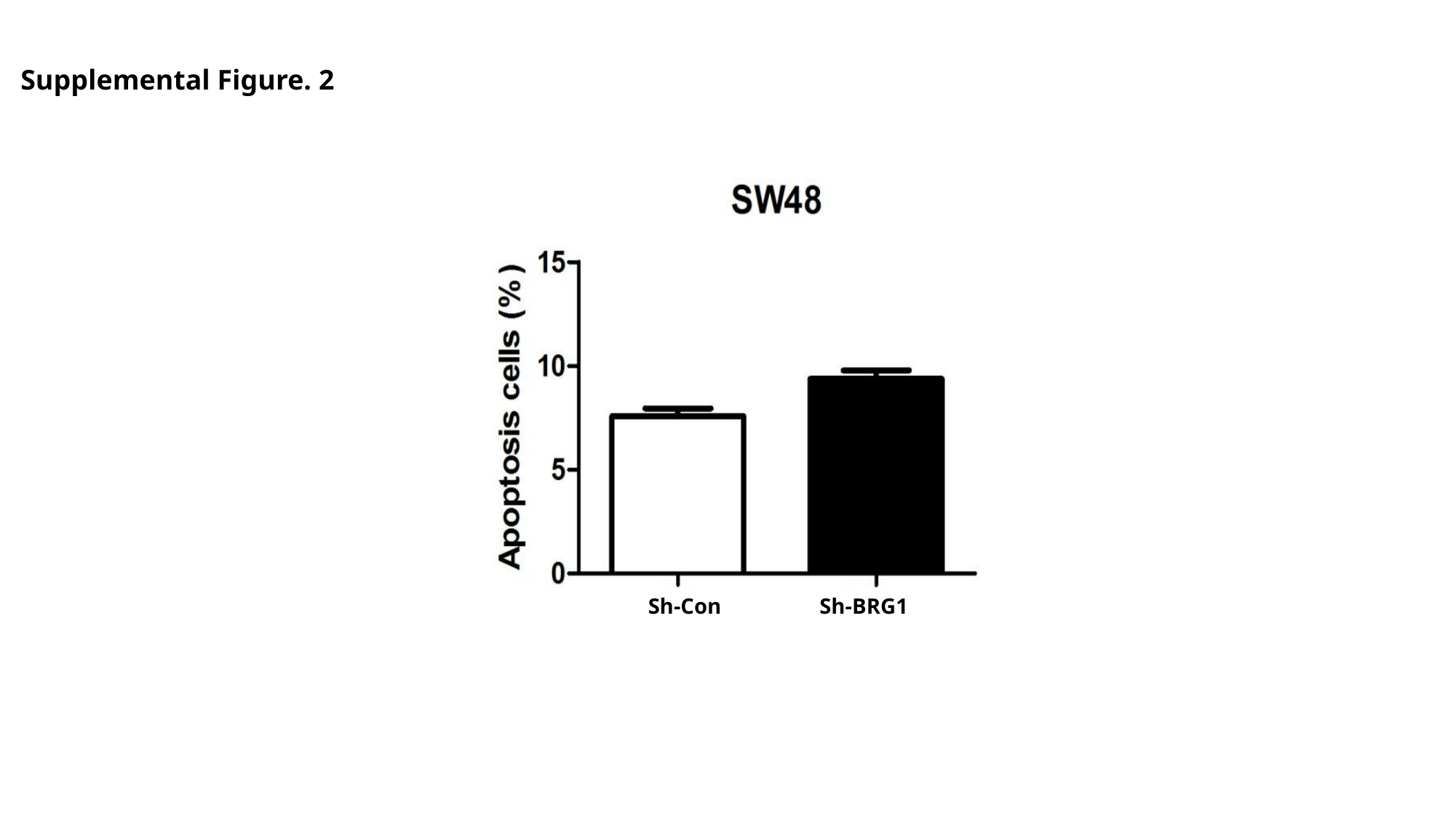

Supplemental Figure. 2
 Sh-Con Sh-BRG1

## Slide 3
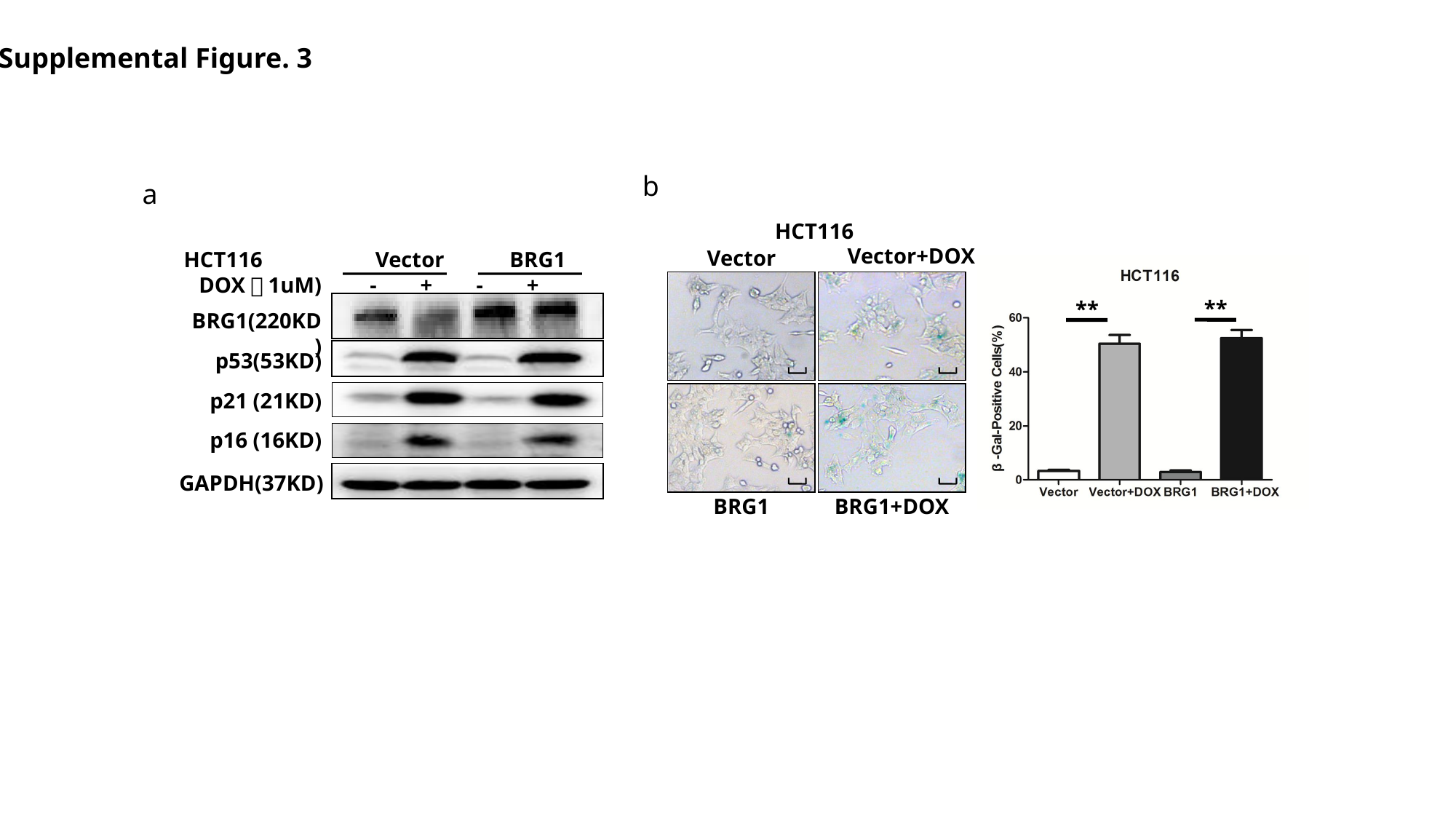

Supplemental Figure. 3
b
a
HCT116
Vector+DOX
Vector
BRG1
BRG1+DOX
**
**
HCT116
 Vector BRG1
DOX（1uM)
 - + - +
BRG1(220KD)
p53(53KD)
p21 (21KD)
p16 (16KD)
GAPDH(37KD)

## Slide 4
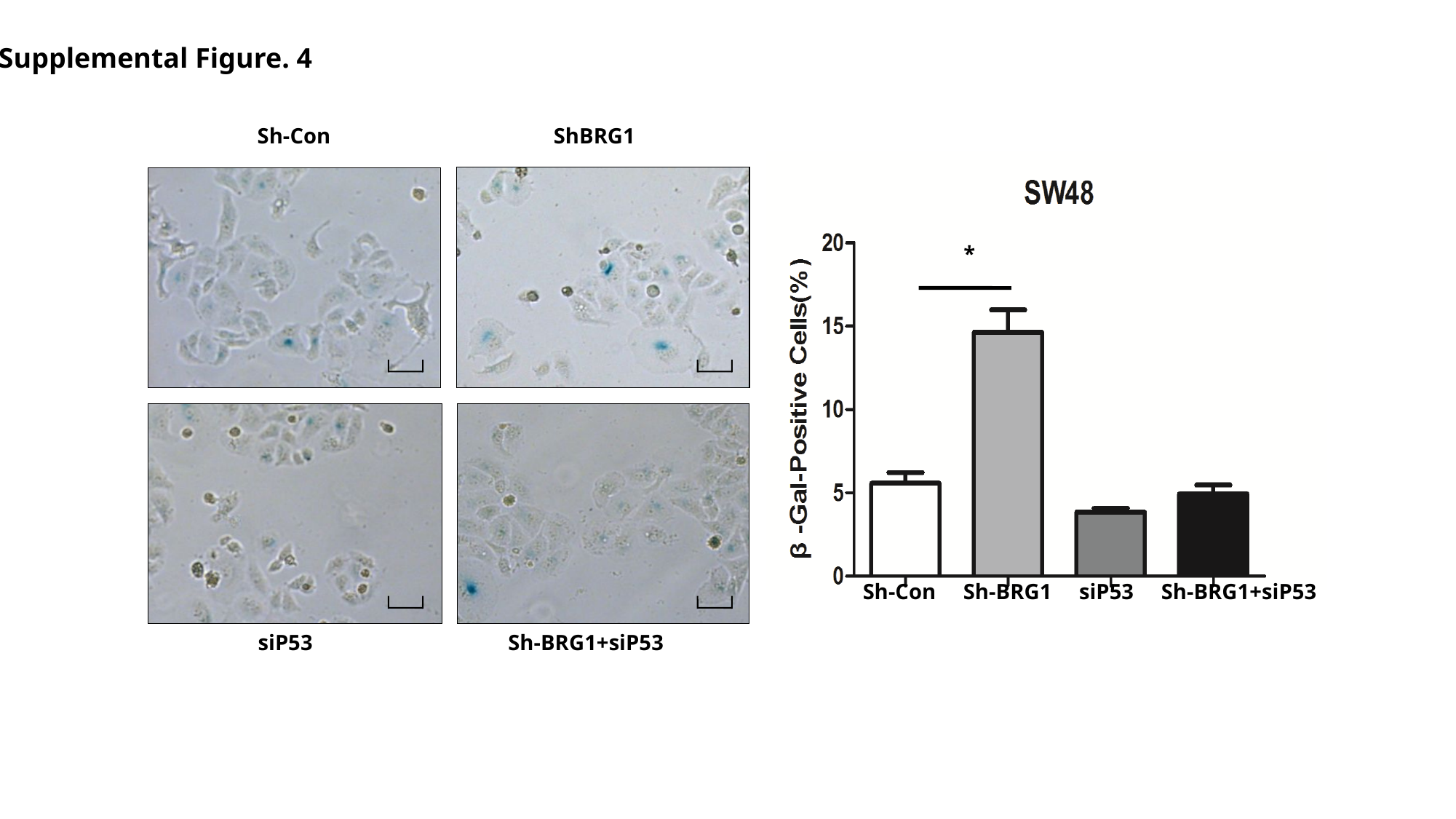

Supplemental Figure. 4
Sh-Con
ShBRG1
siP53
Sh-BRG1+siP53
*
Sh-Con Sh-BRG1 siP53 Sh-BRG1+siP53

## Slide 5
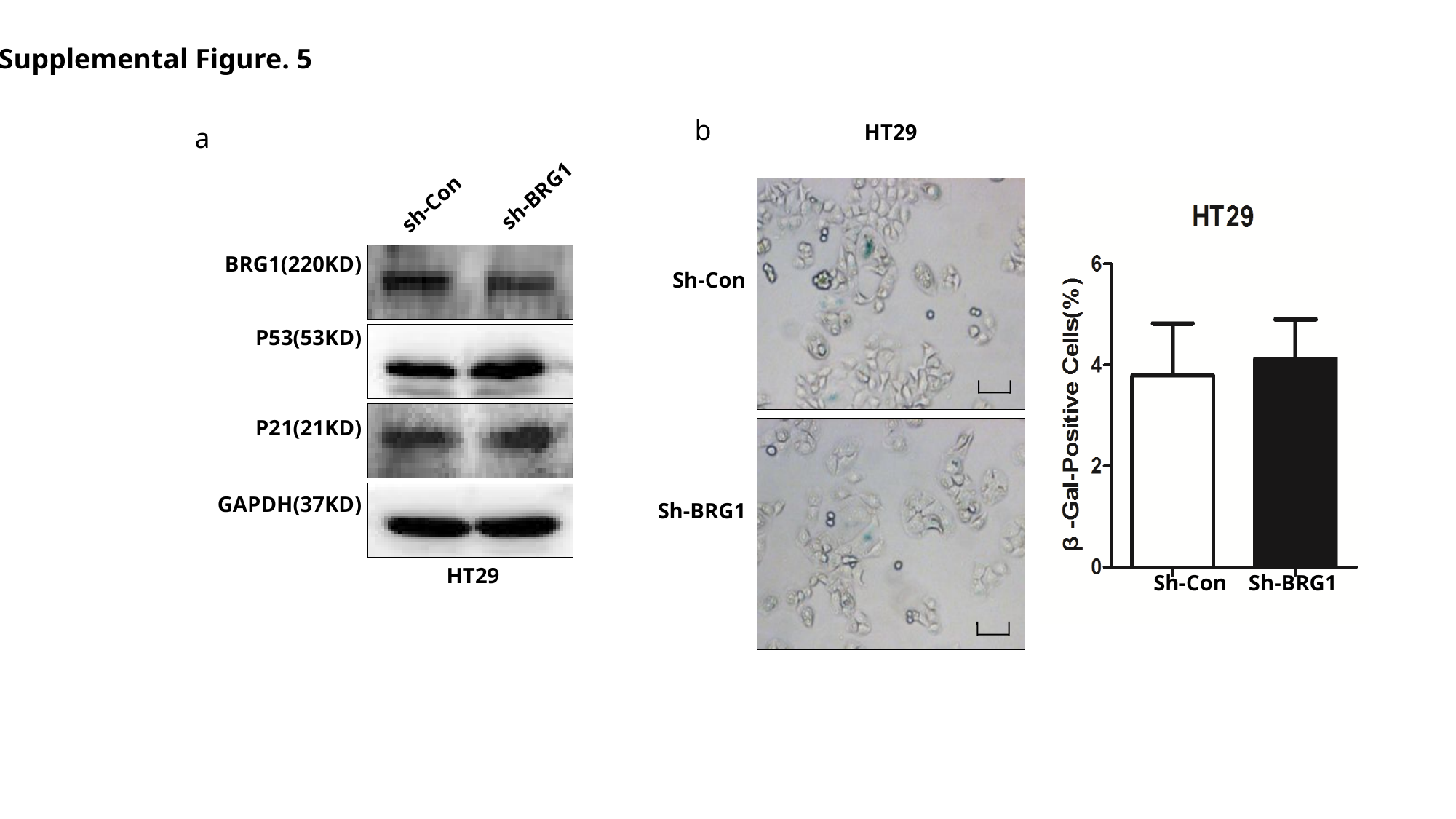

Supplemental Figure. 5
 sh-BRG1
 sh-Con
BRG1(220KD)
P53(53KD)
P21(21KD)
GAPDH(37KD)
HT29
HT29
Sh-Con
Sh-BRG1
Sh-Con Sh-BRG1
b
a

## Slide 6
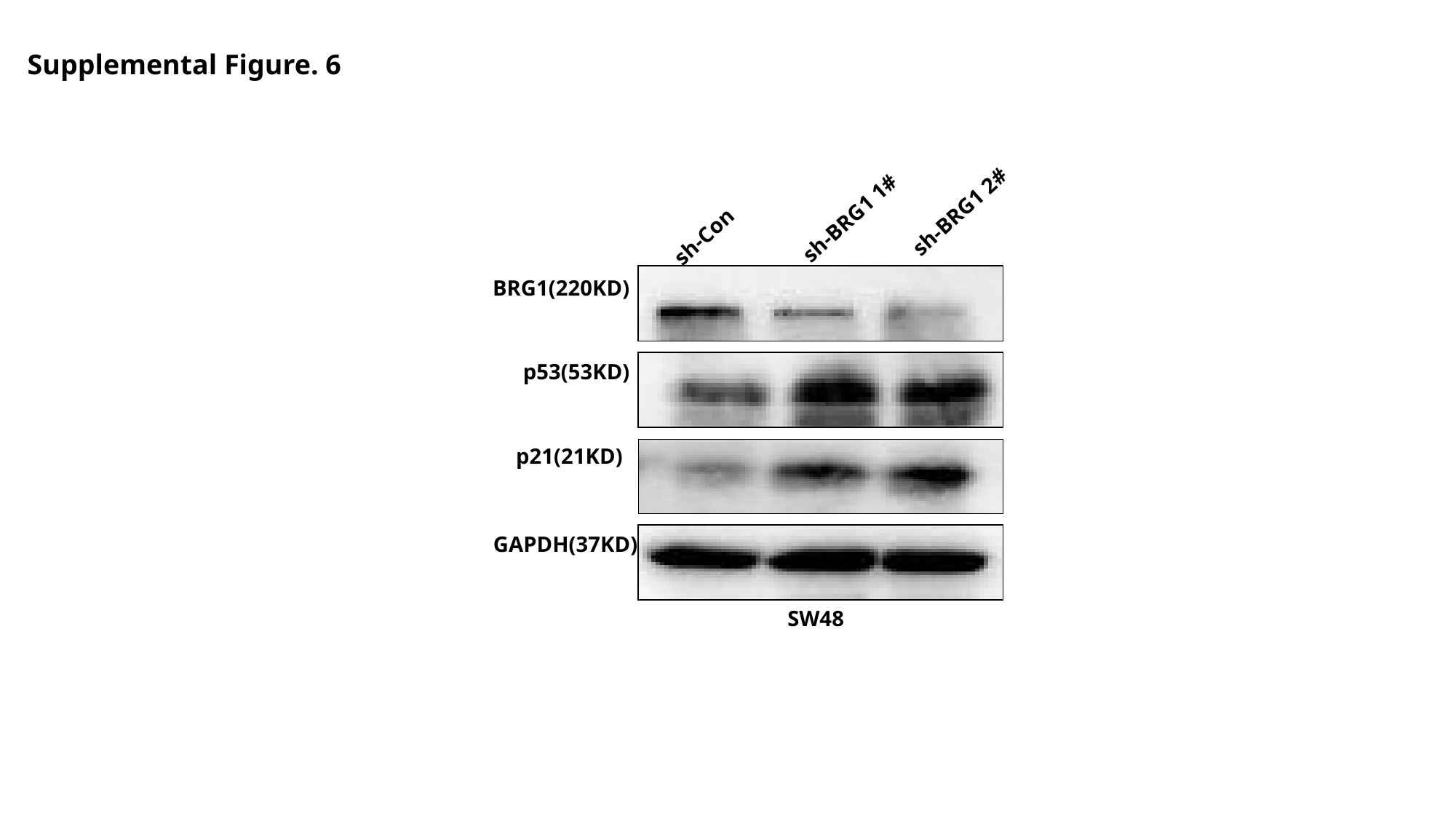

Supplemental Figure. 6
 sh-BRG1 2#
 sh-BRG1 1#
 sh-Con
BRG1(220KD)
p53(53KD)
p21(21KD)
GAPDH(37KD)
SW48

## Slide 7
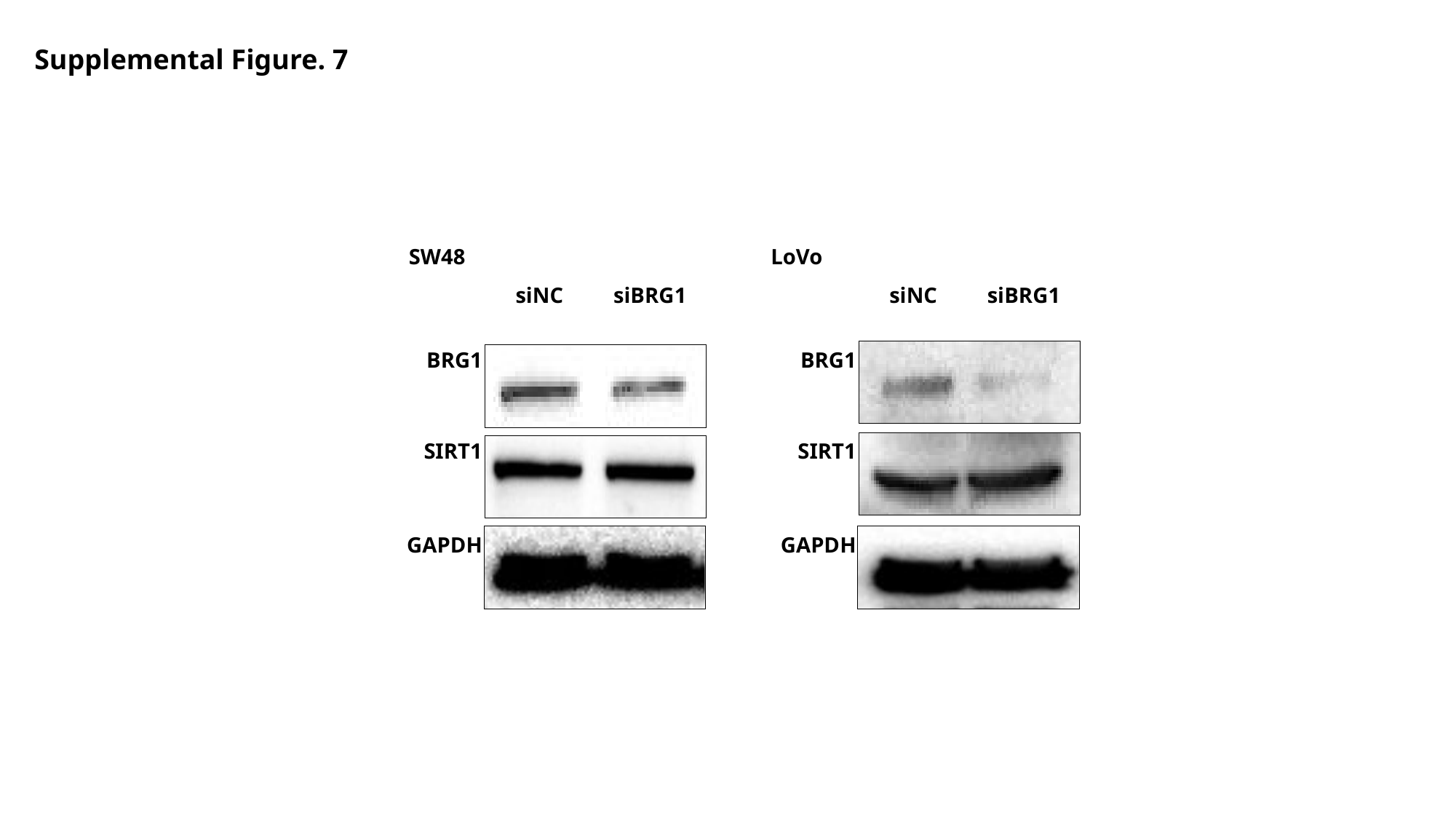

Supplemental Figure. 7
SW48
siNC
siBRG1
BRG1
SIRT1
GAPDH
LoVo
siNC
siBRG1
BRG1
SIRT1
GAPDH

## Slide 8
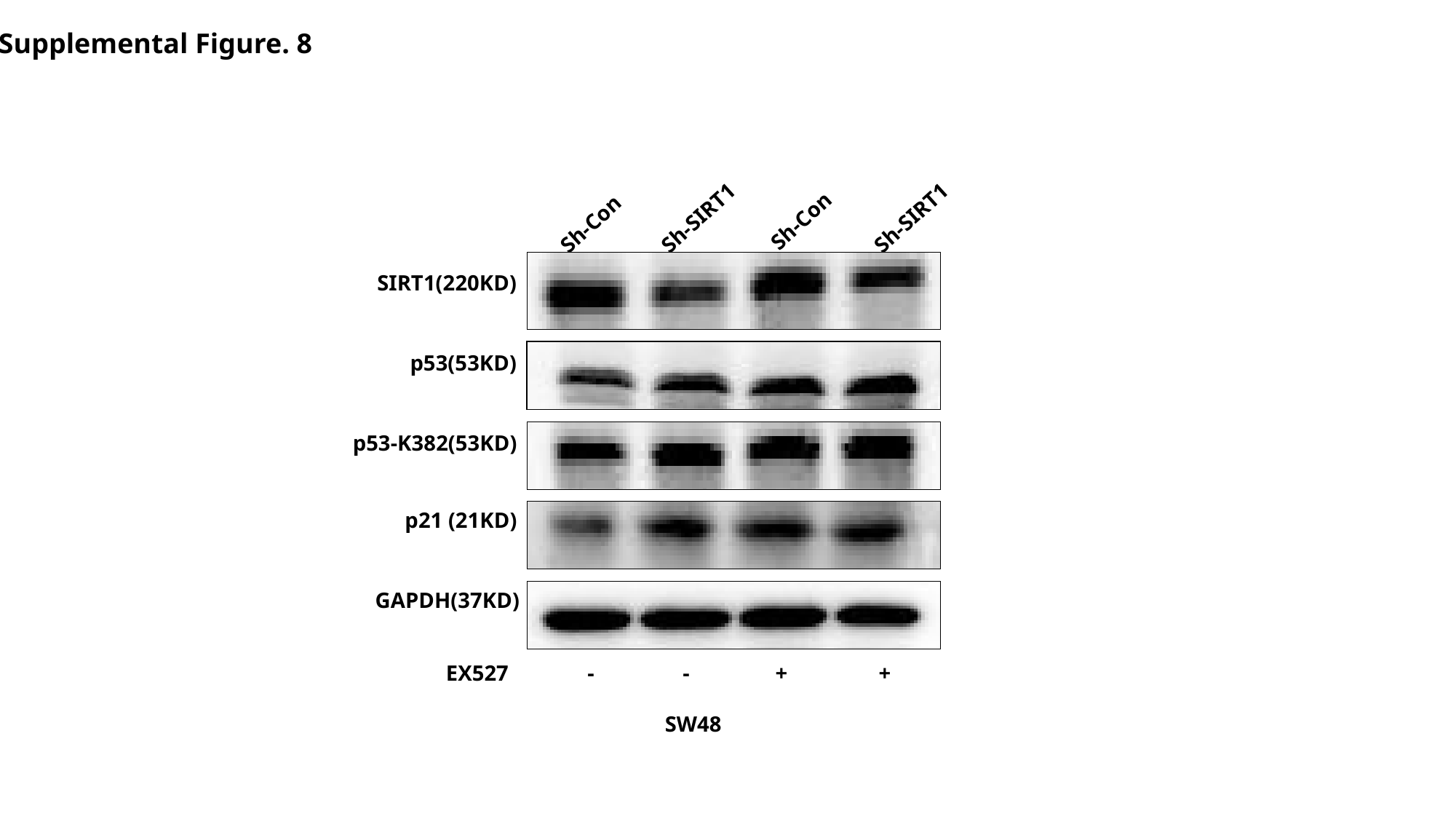

Supplemental Figure. 8
 Sh-Con
 Sh-Con
 Sh-SIRT1
 Sh-SIRT1
SIRT1(220KD)
p53(53KD)
p53-K382(53KD)
p21 (21KD)
GAPDH(37KD)
EX527
-
-
+
+
SW48

## Slide 9
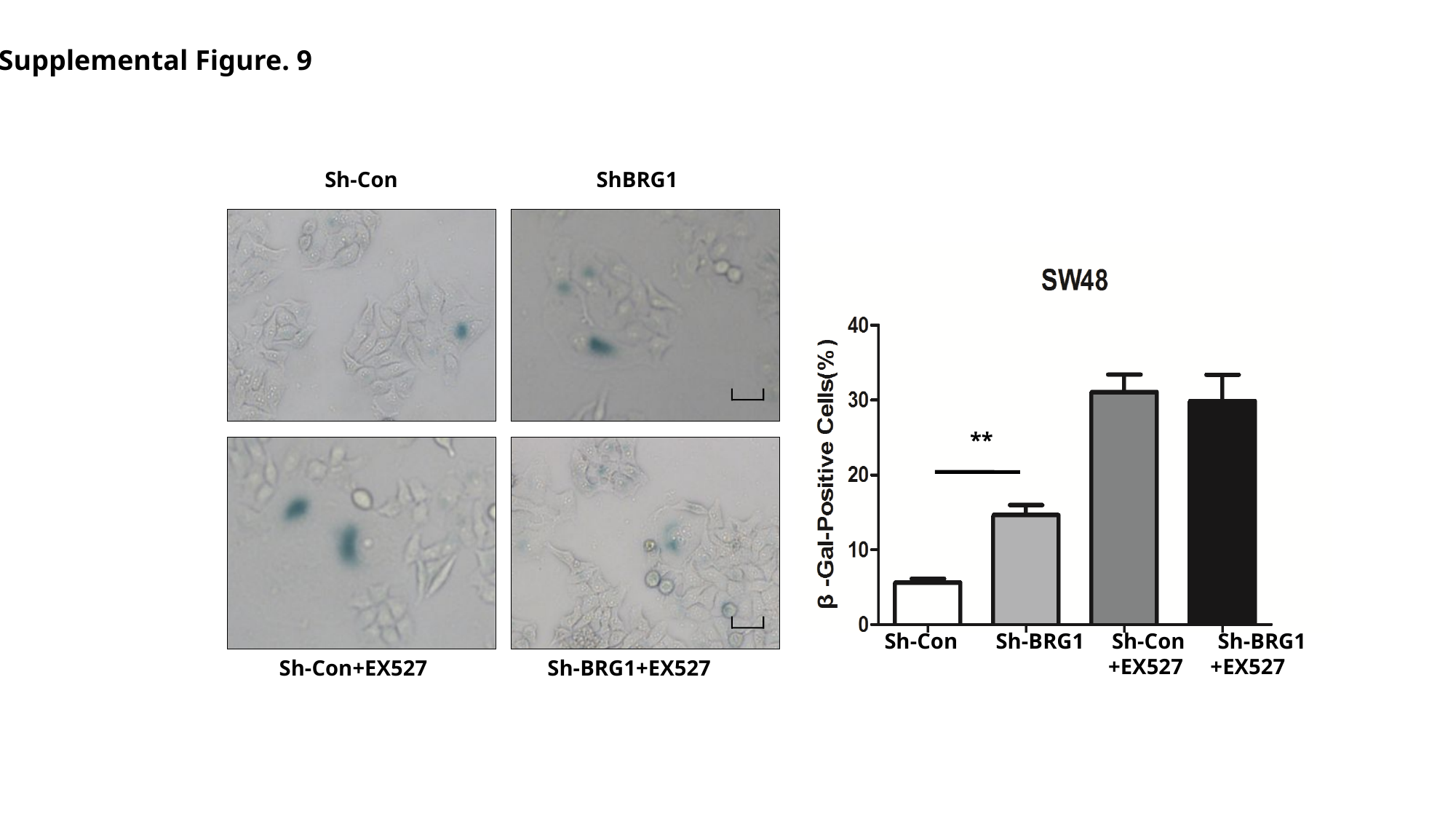

Supplemental Figure. 9
Sh-Con
ShBRG1
Sh-Con Sh-BRG1 Sh-Con Sh-BRG1
 +EX527 +EX527
Sh-Con+EX527
Sh-BRG1+EX527
**
