# Supplementary material for: Loss of BRG1 induces CRC cell senescence by regulating p53/p21 pathway
Source: Cell Death Dis. 2017 Feb 9;8(2):e2607–. doi: 10.1038/cddis.2017.1 (PMC5386468; doi:10.1038/cddis.2017.1)
Supplement: Supplementary Table [file cddis20171x2.docx]

| p53 status in different colon cancer cell lines | | |
| --- | --- | --- |
| HT29 | p.R273H | Missense |
| KM12 | p.V73fs*50 | Frameshift |
| LoVo | WT |  |
| SW480 | p.R273H | Missense |
| SW48 | WT |  |
| SW620 | p.R273H | Missense |
| HCT116 | WT |  |

**Supplemental Table.1 p53 status in different colon cancer cell lines**
