# Supplementary material for: Loss of BRG1 induces CRC cell senescence by regulating p53/p21 pathway
Source: Cell Death Dis. 2017 Feb 9;8(2):e2607–. doi: 10.1038/cddis.2017.1 (PMC5386468; doi:10.1038/cddis.2017.1)
Supplement: Supplementary Information [file cddis20171x3.docx]

**Supplemental Figure Legend**

**Supplemental Figure. 1** SA-β-gal staining (left panel, representative images of SA-β-gal staining, Scale bar：50μm; right panel, percentage of SA-β-gal positive cells from three random microscopic fields) in SW48 Sh-Con and SW48 Sh-BRG1 cells.

**Supplemental Figure. 2** Cell apoptosis analysis in SW48 Sh-Con and SW48 Sh-BRG1 cells (n=3, mean±s.d.).

**Supplemental Figure. 3** (a) Western blot analysis for expression of indicated proteins in HCT116 cells transfected with vector control (vector）or BRG1 plasmid（BRG1）; (b) Cell senescence assay by using SA-β-gal staining (left panel, representative images of SA-β-gal staining, Scale bar：50μm; right panel, average counts of SA-β-gal positive cells from three random microscopic fields) in HCT116 vector and HCT116 BRG1 cells. DOX: doxorubicin (0.25μmol/l, 48 hours).

**Supplemental Figure. 4** SA-β-gal staining (left panel, representative images of SA-β-gal staining, Scale bar：50μm; right panel, percentage of SA-β-gal positive cells from three random microscopic fields) in SW48 Sh-Con and SW48 Sh-BRG1 cells transfected with or without siP53.

**Supplemental Figure. 5** (a) Western blot analysis for expression of indicated proteins in BRG1 knockdown HT29 cells (sh-BRG1) or control cells (Sh-con); (b) SA-β-gal staining (left panel, representative images of SA-β-gal staining, Scale bar：50μm; right panel, percentage of SA-β-gal positive cells from three random microscopic fields) in HT29 Sh-Con and HT29 Sh-BRG1 cells.

**Supplemental Figure. 6** Western blot analysis for expression of indicated proteins in SW48 cells with two different BRG1 knockdown sequence.

**Supplemental Figure. 7** Western blot analysis for expression of indicated proteins in SW48 or LoVo cells transfected with SiRNA control(Si-NC) or SiRNA BRG1(Si-BRG1).

**Supplemental Figure. 8** Western blot analysis for expression of indicated proteins in SW48 cells with different condition.

**Supplemental Figure. 9** SA-β-gal staining (left panel, representative images of SA-β-gal staining, Scale bar：50μm; right panel, percentage of SA-β-gal positive cells from three random microscopic fields) in SW48 cells in labeled treatment .
